# Cinnamon extract supplementation improves inflammation and oxidative stress induced by acrylamide: An experimental animal study 

**Published:** 2020

**Authors:** Fatemeh Haidari, Majid Mohammadshahi, Behnaz Abiri, Mehdi Zarei, Mojdeh Fathi

**Affiliations:** 1 *Nutrition and Metabolic Diseases Research Center,* *Department of Nutrition, Ahvaz Jundishapur University of Medical Sciences, Ahvaz, Iran*; 2 *Hyperlipidemia Research Center, Department of Nutrition, Ahvaz Jundishapur University of Medical Sciences, Ahvaz, Iran*; 3 *Department of Nutrition, Faculty of Paramedicine, Ahvaz Jundishapur University of Medical Sciences, Ahvaz, Iran*; 4 *Department of Food Hygiene, Faculty of Veterinary Medicine, Shahid Chamran University of Ahvaz, Ahvaz, Iran*; 5 *Department of Nutrition, Faculty of Paramedicine, Ahvaz Jundishapur University of Medical Sciences, Ahvaz, Iran*

**Keywords:** Cinnamon extract, Acrylamide, Oxidative stress, Liver enzyme, Inflammation

## Abstract

**Objective:**

Toxic effects of acrylamide on body organs incline researches to prevent or decrease these effects. The objective of the present study was to evaluate the effects of cinnamon extract (CE) supplementation on inflammation and oxidative stress induced by acrylamide in rats.

**Materials and Methods:**

Thirty two rats were divided into four groups as follow 1) The control group received distilled water, 2) Acrylamide- intoxicated group was administrated with 35 ml/kg/day acrylamide for two weeks, 3) Acrylamide- intoxicated rats treated with CE 250 mg/kg/day for 28 days, and 4) Acrylamide- intoxicated rats treated with CE 500 mg/kg day for 28 days. Fasting blood sample was obtained for subsequent analysis.

**Results:**

The results showed that acrylamide- intoxicated group had significantly higher levels of malondialdehyde, tumor necrosis factor alpha, high sensitive C reactive protein, leptin and alanine transaminase (p<0.05 to p<0.01) and lower levels of total antioxidant capacity compared to the control group; whereas, cinnamon extract administration remedied levels of total antioxidant capacity, malondialdehyde, tumor necrosis factor alpha, high sensitive C reactive protein and leptin in the treatment groups, but it did not have a significant effect on adiponectin and liver enzymes (p>0.05).

**Conclusion:**

This study suggests that cinnamon extract may potentially be effective as a dietary source of bioactive compounds for managing acrylamide intoxication.

## Introduction

The liver is an essential organ for xenobiotics detoxification, which are delivered from the environment, drugs, alcohol and foods to the body (Mroueh et al., 2004 ▶). One of the components that has a toxic effect on the liver is acrylamide (AA) (Ansar et al., 2016 ▶). Although AA has industrial applications, it is also formed during Millard reaction in backed and fried foods. AA is a responsible for desirable flavor and color in fried foods (Lasekan and Abbas, 2010 ▶). Moreover, one of the most common sources of exposure to AA is cigarette and tobacco (Papoušek et al., 2014 ▶).

AA is a reactive component that is metabolized in the liver and changed to glycidamide (Taubert et al., 2006 ▶). Both AA and its metabolite are destructive (Hansen et al., 2010 ▶). Oxidative stress is a result of an imbalance between the production of free radicals and the capacity of antioxidant systems. Detoxification of AA in the liver causes depletion of glutathione source and decreases antioxidant enzymes and generally causes oxidative stress in the liver and other organs (Watzek et al., 2013 ▶). Destruction of liver tissue and release of aminotransferase enzymes result in AA-induced oxidative stress (Zhao et al., 2015 ▶). Besides, previous studies have demonstrated that oxidative stress stimulates inflammatory pathways and results in increased levels of cytokines and some of the adipocytokines such as leptin and adionectin (Septembre‐Malaterre et al., 2016 ▶). Therefore, a previous study indicated that AA induces inflammation (Alturfan et al., 2012 ▶). Neurotoxicity, reproductive toxicity and genotoxicity are the most obvious complications of AA observed in animal and human studies (Liu et al., 2015 ▶; Jiang et al., 2007 ▶; Goffeng et al., 2008 ▶).

Following the growth of fast foods and the smoking industry in the word, the global concern about AA intake has recently increased (Braithwaite et al., 2014 ▶). The Tobacco Atlas in 2013 reported that Eastern and South-Eastern Asia and Eastern Europe have the highest male smoker prevalence (Einstein et al., 1935 ▶). 

The effectiveness of present synthetic medications in the treatment of liver diseases is not satisfactory and these chemicals show undesirable side effects. Hence, many phytochemicals and medicinal herbs, as alternative and complementary medications, have been evaluated in liver diseases (Ghobadi Pour et al., 2019 ▶). Among these medicinal herbs, cinnamon extract (CE) has shown hepato-protective impacts. *Cinnamomum zeylanicum *(CZ) is a plant species and used as spice and tea in different cultures. CZ has many bioactive components such as flavonoids, tannins, terpenoids, glycosids and alkaloids (Shihabudeen et al., 2011 ▶). Essential oils of the bark of CZ contain three main components including trans-cinnamaldehyde, eugenol, and linalool (El-Baroty et al., 2010 ▶). Previous studies suggested anti-inflammatory, antioxidant, anticancer and hepato-protective properties of CZ (Hagenlocher et al., 2016 ▶; Roussel et al., 2009 ▶; Zhang et al., 2016 ▶; Eidi et al., 2012 ▶).

Therefore, recently, the use of CZ as a herbal medicine has received attention. The aim of the present study was to investigate the effect of cinnamon extract (CE) on hepatotoxicity and changes in levels of adipocytokines induced by AA in rats. 

## Materials and Methods

**Animals**

Thirty two male Wistar rats (6-8 weeks old and body weight 150-200 g) were purchased from Physiology Research Center of Ahvaz University of Medical Sciences. The animals were acclimatized in a quite controlled animal room (22±3°C), with 55±5% humidity, and 12-hour light/dark cycles. Animals had free access to standard pellet diet and water. The study was approved by and performed under the guidelines of the Research Ethics Committee of Ahvaz Jundishapur University of Medical Sciences, Iran (NRC-9414).

**Experimental protocol**

After the acclimatization period (2 weeks), the animals were randomly divided into four groups (n=8 in each group) as follows: Group 1: Control rats (sham) received distilled water, Group 2: acrylamide- intoxicated rats, Group 3: acrylamide- intoxicated rats treated with CE 250 mg/kg/day, Group 4: acrylamide- intoxicated rats treated with CE 500 mg/kg/day. 

For inducing intoxication, groups 2, 3 and 4 received 35 mg/kg/day AA (800830, Merck, Germany), dissolved in distilled water and administered through gastric gavage for 2 weeks (El-Mehi and El-Sherif, 2015 ▶; Ghorbel et al., 2017 ▶). Control group received the same amount of distilled water.

**Extraction methods**

Cinnamon barks were purchased from local market, identified and authenticated by an expert from Ahvaz Shahid Chamran University. The extract preparation was performed based on Gaique et al. study (Gaique et al., 2015 ▶). Dried cinnamon barks were washed in distilled water, dried and ground to powder. Ten grams of fine powder was solved in 100 ml distilled water in covered Erlenmeyer flask and incubated at 60°C for 1 hr. The extract was centrifuged at 1000 *g *for 5 min at 4^∘^C. Supernatant in a clean bottle was stored at -20°C (Gaique et al., 2016 ▶). The aqueous extract was administrated orally via gavage tubes for 4 weeks.

**Preparation of serum and liver samples**

Twenty-four hours after the final CE exposure, the rats were anesthetized using diethyl ether and sacrificed and fasting blood samples were collected directly from the heart. Blood samples were then centrifuged at 4000 g for 10 min, and sera were kept at -70°C for posterior biochemical analysis. The liver of animals was also excised, weighed and rapidly perfused with cold saline (0.9%) and then, placed in chilled KCL (1.15% KCl w/v) containing 0.1 mM EDTA. The livers were then chopped into 4-5 volumes of 50 mM phosphate buffer (pH 7.4) and homogenized using a homogenizer. The homogenate was then centrifuged at 3000 g for 10 min, the above lipid layer was carefully removed and the resulting supernatant fraction was further centrifuged at 12,000 g for 60 min at 4°C. The supernatant was stocked at -70°C until assayed (Haidari et al., 2013 ▶).

**Assessment of the biochemical parameters**

Total antioxidant capacity (TAC), tumor necrosis factor alpha (TNF-α), high-sensitivity C-reactive protein (hs-CRP), leptin and adiponectin were quantified using ELISA method by standard kits (Randox labs- UK for TAC; Orgenium laboratories-Finland for TNF-α; Labor Diagnostika Nord for hs-CRP; Boster- China for leptin and adiponectin) according to the manufacturer's instructions. The concentration of malondialdehyde (MDA) was assayed for lipid peroxidation assessment spectrophotometrically, based on the reaction of MDA with thiobarbituric acid. Serum analysis of marker enzymes, such as the activities of AST, ALT and ALP was performed using commercial kits (Pars- azmoon, Iran).

**Statistical analysis**

The normality of distribution was determined using the Kolmogorov-Smirnov test. Data were subjected to one-way analysis of variance (ANOVA) followed by Tukey test. SPSS version 17.0 was used for statistical analysis of the data. The levels of significance were considered less than 0.05. All the data are expressed as mean±standard deviation (SD).

## Results

**Effect of CE administration on serum and liver oxidative stress**

The mean of the serum and liver levels of TAC and MDA at the end of the study are presented in Table 1. The results showed that the AA- intoxicated group had significantly lower TAC levels in both serum and liver samples compared to the control group (p=0.005 and 0.002, respectively). In contrast, AA- intoxicated group had significantly higher MDA levels in both serum and liver samples in comparison to the control group (p=0.05 and 0.021, respectively). The administration of CE at two doses significantly increased serum and liver TAC levels (p<0.05 to p<0.01) and decreased both liver and serum MDA compared to the AA- intoxicated group (p<0.01 for all cases).

**Table 1:** Effect of CE administration on serum and liver oxidative stress biomarkers.^a^

**Groups**	**Serum TAC ** **(nmol/l)**	**Liver TAC (U/g)**	**Serum MDA (μmol/l)**	**Liver MDA ** **(nmol /mg)**
Control	9.46±0.58	9.73±0.81	5.24±2.29	4.95±2.45
Acrylamide intoxicated	7.88±1.17^##^	7.86±1.00^##^	8.72±3.24^#^	9.28±4.13^#^
Acrylamide+CE (250 mg/kg/day)	9.35±1.07^*^	7.86±1.18	4.36±.31^**^	3.68±.87^**^
Acrylamide+CE (500 mg/kg/day)	9.64±0.42^**^	9.07±.37^*^	3.53±.99^**^	4.05±.88^**^

^a^ All values are expressed as mean±SD (n=8). ANOVA followed by Tukey test was used for statistical analysis. * indicates p<0.05 and ** indicates p<0.01 *vs.* acrylamide intoxicated group; # indicates p<0.05 and ^##^ indicates p<0.01 *vs.* control group.

TAC: total antioxidant capacity; MDA: malondialdehyde; CE: cinnamon extract.

**Effect of CE administration on serum levels of leptin, adiponectin, and inflammatory biomarkers**

Table 2 shows the effect of AA and various dosages of CE on serum levels of leptin, adiponectin, and inflammatory biomarkers. The results showed that AA increased levels of TNF-α, hs-CRP and leptin in the AA- intoxicated group compared to the control group (p=0.021, 0.002 and 0.005, respectively). CE administration, in both doses, led to a significant decrease in the serum levels of TNF-α, hs-CRP and leptin compared to the AA- intoxicated group (p<0.05 to p<0.01). However, it was not significant for hs-CRP in treatment group with 250 mg/kg CE (p=0.055). The present study showed that intoxication with AA does not significantly change adiponectin levels in AA- intoxicated group (p=0.145). Consequently, CE administration did not change the serum levels of adiponectin in treatment groups.

**Table 2:** Effect of CE administration on serum levels of leptin, adiponectin, and inflammatory biomarkers. ^a^

**Groups**	**TNF-α** ** (** **pg/ml** **)**	**hs-CRP (mg/l)**	**Leptin (ng/ml)**	**Adiponectin** ** (μg/ml)**
Control	31.13±13.72	631.37±111.64	4.41 ± 0.48	15.24±3.65
Acrylamide intoxicated	56.13±18.61^#^	895.25±82.90^##^	6.41 ± 1.28^##^	11.30±6.04
Acrylamide + CE (250 mg/kg/day)	22.00±10.24^**^	697.00±245.98	4.03±1.07^**^	16.89±3.51
Acrylamide + CE (500 mg/kg/day)	32.70±17.19^*^	664.50±89.06^**^	4.60±0.74^**^	15.05±6.25

^a^ All values are expressed as mean±SD (n=8). ANOVA followed by Tukey test was used for statistical analysis. * indicates p<0.05 and ** indicates p<0.01 *vs.* acrylamide intoxicated group; # indicates p<0.05 and ^##^ indicates p<0.01 *vs.* control group.

TNF-α: tumor necrosis factor α; hs-CRP: high sensitive C-reactive protein; CE: cinnamon extract.

**Effect of CE administration on liver enzymes activities**

The effect of CE administration on liver enzymes activities is summarized in Table 3. The results revealed that AA intoxication led to a significant increase in ALT activity (p=0.041) in the AA- intoxicated group compared to the control group. In this study, AA did not significantly change AST and ALP activities in the AA- intoxicated group compared to the control group. Administration of CE did not significantly affect these enzymes activity (p>0.05).

**Table 3:** Effect of CE administration on liver enzymes activities. ^a^

**Groups**	**ALT ** **(U/dl)**	**AST ** **(U/dl)**	**ALP ** **(U/dl)**
Control	63.00±17.22	218.42±23.45	632.71±188.09
Acrylamide intoxicated	81.12±13.76^#^	204.37±80.93	645.50±244.28
Acrylamide + CE (250 mg/kg/day)	65.62±40.18	143.00±66.15	647.00±266.07
Acrylamide + CE (500 mg/kg/day)	76.50±22.69	153.12±62.75	781.25±155.04

^a^ All values are expressed as mean±SD (n=8). ANOVA followed by Tukey test was used for statistical analysis. # indicates p<0.05 *vs.* control group.

ALT: alanine transaminase; AST: aspartate transaminase; ALP: Alkaline phosphatase; CE: cinnamon extract.

## Discussion

Because of the increasing prevalence of hepatic diseases and also exposure to AA in the world, prevention or decrement of the harmful effects of AA in body, is necessary (Williams et al., 2011 ▶). Previous studies demonstrated the antioxidant and anti-inflammatory properties of components of CZ (Gunawardena et al., 2015 ▶). Furthermore, previous studies demonstrated that CE has hepato-protective effects and leads to decreases in aminotransferase enzymes (Moselhy and Ali., 2009 ▶). In the present study, the effect of CE on experimental liver damage and changes of adipocytokines status induced by AA, was investigated. The current study revealed that CE significantly suppressed oxidative stress status induced by AA. The serum and liver levels of TAC and MAD were improved in the treatment groups. Previous studies indicated that antioxidant effects of cinnamon are mediated through elevating antioxidant enzymes, increasing the source of glutathione and decreasing lipid peroxidation (Dehghan et al., 2014 ▶). Roussel et al. showed the antioxidant effects of cinnamon extract in obese humans with impaired fasting glucose (Roussel et al., 2009 ▶). Trans-cinnamaldehyde is the most important bioactive component of the essential oil of CZ bark (El-Baroty et al., 2010 ▶). Many studies showed antioxidant properties of cinnamaldehyde (Wang et al., 2015 ▶). Abd El-Raouf suggested that antioxidant and anti-inflammatory properties of cinnamic acid and cinnamaldehyde lead to a protective effect on cisplatin (CP)-induced splenotoxicity in rats (El‐Raouf et al., 2015 ▶). Wang et al. revealed that antioxidant activity of cinnamaldehyde and its protective effect on endothelial dysfunction in high glucose conditions is mediated through activating NF-E2-related factor 2 (Nrf2) and up-regulation of the downstream target proteins. It is shown, cardiovascular protective effects of cinnamaldehyde (Wang et al., 2015 ▶). 

Cao et al. revealed that cinnamon polyphenol extract increased TTP (a phosphorylated protein that down-regulates pro-inflammatory cytokines) expression. However, cinnamon polyphenol extract also increased the expression of pro-inflammatory cytokines such as TNFα, IL-6 and COX-2 (cyclooxigenase-2). The study suggested that net elevated levels of TTP were greater than pro-inflammatory cytokines and generally cinnamon polyphenol extract has anti-inflammatory effects (Cao et al., 2007 ▶). Another *in vivo* and *in vitro* study indicated that cinnamon aqueous extract decreased levels of TNFα and IL-6 in an LPS-induced model; this study suggested that this effect resulted from polyphenols of cinnamon. In the present study, CE decreased serum levels of TNF-α and hs-CRP (Hong et al., 2012 ▶). 

Leptin and adiponectin are two major adipocytokines and have a role in the inflammatory process. Leptin and adiponectin stimulate pro-inflammatory and anti-inflammatory pathways, respectively (Shen et al., 2009 ▶; Ohashi et al., 2010 ▶). Moreover, leptin and adiponectin showed protective effect on the nonalcoholic fatty liver disease, respectively (Machado et al., 2012 ▶; Matsunami et al., 2010 ▶). In this study, serum levels of leptin significantly increased in the AA- intoxicated group compared to the control group. Thus, elevated leptin levels can play a role in increment of TNF-α and hs-CRP levels in intoxicated rats. Septembre-Malaterre et al. reported *Curcuma longa* polyphenols through decreasing oxidative stress and TNF-α, IL-6 and nuclear factor κappa B, and increasing adiponectin secretion, ameliorate obesity-related metabolic disorders (Septembre‐Malaterre et al., 2016 ▶). 

Shatwan et al. showed that CE administration decreased leptin levels and appetite in obese rats that fed with high fat diet (Shatwan et al., 2013 ▶). Neveen also administered 100 and 200 mg/kg/day cinnamon aqueous extract to diabetic obese rats for 6 weeks. The study suggested that decrement of leptin levels results in decreased adipose size in diabetic obese rats treated with cinnamon aqueous extract (Ismail NS, 2014 ▶). The current study showed that CE at both 250 and 500 mg/kg concentrations, decreased levels of leptin in the treatment groups. Oxidative stress leads to cytokines and adipokines dysregulation, including increasing levels of leptin, TNF-α and hs-CRP and decreasing transcription of adiponectin, (Ejaz et al., 2009 ▶; Tang et al., 2012 ▶). This study suggests that improvement of the levels of inflammatory markers resulted in suppression of oxidative stress by antioxidant components of CZ. It was indicated that flavonoids, anthraquinone, glycosides, alkaloids, steroids, tannins and terpenoids are present in cinnamon barks (Shihabudeen et al., 2011 ▶). Some of phytochemicals such as flavonoids, triterpenoids, saponins and alkaloids have shown a hepatoprotective activity (Kokanova-Nedialkova et al., 2016 ▶; Wang et al., 2016 ▶; Zheng et al., 2015 ▶; Raj et al., 2010 ▶). 

Although previous studies reported the modulatory effect of polyphenols on adiponectin levels, but in the present study, CE did not alter adiponectin levels in the treated rats (Heber et al., 2014 ▶). Normal levels of adiponectin in AA- intoxicated rats may result in this contradiction (Mahmoud AM, 2013 ▶; Li et al., 2006 ▶). It is proposed to use higher doses of CE on adiponectin levels. Previous studies reported increased levels of ALT, AST and ALP in AA intoxication (Ansar et al., 2016 ▶; Watzek et al., 2013 ▶; Alturfan et al., 2012 ▶). These studies proposed that AA through liver destruction, elevates aminotransferase levels. The present study showed a significant effect of AA on increasing ALT activity in the AA- intoxicated group compared to the normal state. The present study suggests that AA induced oxidative stress and inflammation and damaged hepatic cell membranes thus causing increased ALT activity. Indeed, direct and indirect contribution of elevated levels of leptin to liver injury process may result in increased ALT activity. The two doses of CE did not alter the ALT activity levels compared to the non-treated group. Similar results were reported previously (Koochaksaraie et al., 2011 ▶).

Wickenberg et al. in 2014 ▶ showed that administration of 6 g cinnamon twice a day for 12 weeks had no significant impact on aminotransferase enzyme in diabetic patients (Wickenberg et al., 2014 ▶). Lue et al. reported that cinnamon supplementation at low and high doses for 3 months did not change liver enzymes in diabetic patients ( Lu et al., 2012 ▶). 

However, numerous studies suggested the beneficial effect of cinnamon on aminotransferase enzymes (Askari et al., 2014 ▶). Kamal et al. showed that CE ameliorated elevated levels of ALT and AST in hypercholesterolimic rats (Amin and El-Twab, 2009 ▶). Treatment with CE significantly remedied the impact of CCL4 on liver aminotransferases (Moselhy and Ali, 2009 ▶). Unlike a previous study, AA intoxication did not affect AST and ALP activity in this study. Differences in dosage, type and period of administration may led to this difference. However, it is indicated that the elevated ALT among all aminotransferases is an illustrator of liver injury. Furthermore, CE did not alter AST and ALP activity. Thus, it is suggested that CE does not have any significant effect on levels of enzyme activity in normal and injury liver state. 

The present study provides evidence that oral administration of CE partially improved liver injury in AA-intoxicated rats by decreasing inflammatory factors. It is proposed that the mechanism of cinnamon extract action is probably mediated through scavenging free radicals and ameliorating inflammatory biomarkers.
